# Nose-to-Eye Delivery: The Potential of Intranasal Administration in Ophthalmology

**DOI:** 10.3390/jcm15135029

**Published:** 2026-06-27

**Authors:** Maria Letizia Adezio, Danilo Iannetta, Gianluca Manni, Giacomo Visioli, Gloria Roberti, Ludovico Alisi

**Affiliations:** 1 Department of Sense Organs, Sapienza University of Rome, 00185 Rome, Italy; marialetizia.adezio@uniroma1.it (M.L.A.); danilo.iannetta@uniroma1.it (D.I.); gianluca.manni@uniroma1.it (G.M.); giacomo.visioli@uniroma1.it (G.V.); gloria.roberti@uniroma1.it (G.R.); 2 IRCCS—Fondazione Bietti, 00184 Rome, Italy; 3 Ophtalmology Unit, San Giovanni Addolorata Hospital, 00184 Rome, Italy

**Keywords:** intranasal, drug delivery, ocular bioavailability, nasolacrimal reflex, posterior segment, neuroprotection, blood–retinal barrier

## Abstract

Non-invasive drug delivery for ocular diseases remains a significant challenge in ophthalmology, as conventional eye drops offer less than 5% bioavailability due to pre-corneal barriers and the corneal epithelium. This review explores the intranasal (IN) route as a promising strategy for targeting both the anterior and posterior segments of the eye. The IN route leverages several distinct pathways: the nasolacrimal reflex for remote physiological stimulation; the “neural bridge” through the cribriform plate, allowing direct perineural and vascular transport via the olfactory and trigeminal nerves to bypass the blood–retinal barrier; and systemic absorption that avoids hepatic first-pass metabolism. Pre-clinical evidence indicates that IN administration of agents such as erythropoietin, nerve growth factor, and insulin achieves superior retinal concentrations compared to topical or systemic dosing, offering neuroprotection in models of retinal degeneration and glaucoma. Clinically, varenicline nasal spray is already FDA-approved for dry eye disease, while intranasal steroids demonstrate a favorable ocular safety profile without significantly increasing intraocular pressure. Although limited by mucociliary clearance and small delivery volumes, the IN route offers a painless, non-invasive alternative to intraocular injections, potentially enhancing patient compliance. Future advancements in mucoadhesive nanocarriers are essential to optimize drug residence time and realize the full potential of nose-to-eye delivery in chronic ophthalmic care.

## 1. Introduction: The Challenges of Topical Administration

While topical drug delivery via conventional eye drops remains the standard of care due to its non-invasiveness, its efficacy is severely compromised by physiological and pharmacological barriers, especially when targeting the posterior segment.

### 1.1. Low Pre-Corneal Ocular Bioavailability

A primary challenge in topical delivery is the limited fraction of the drug effectively retained within the pre-corneal tear film. It is estimated that less than 5% of the topically applied dose remains available for absorption [1].

When a standard 50 μL droplet is administered via a conventional commercial dispenser, the total pre-corneal tear volume increases from a baseline of 7 μL to only 10 μL (in an upright patient during blinking). Consequently, a maximum of 20% of the administered dose is initially retained (10 μL/50 μL). Furthermore, the tear film undergoes rapid fluid turnover, estimated at 16% per minute in the resting eye. This rate accelerates significantly if the formulation induces reflex lacrimation and increased blinking frequency, both of which facilitate rapid drainage [2]. These dynamics drastically reduce the ocular residence time—the duration of drug contact with the corneal and conjunctival epithelia. As a result, it is estimated that only 10% of the initial dose persists on the ocular surface after 4 min, dwindling to a mere 3.4% after 10 min [2].

### 1.2. Ocular Barriers and Permeability

To reach internal ocular tissues, pharmacological agents must cross the cornea, which serves as a highly effective protective barrier characterized by tight junctions and a complex three-layered structure: a lipophilic outer epithelium, a hydrophilic central stroma, and a lipophilic inner endothelium. Consequently, only drugs with an optimal hydrophilic-lipophilic balance (amphiphilic drugs) can effectively permeate this barrier.

Several pharmacological parameters influence corneal drug penetration, including drug concentration, solubility, viscosity, lipophilicity, pH, molecular weight, the choice of vehicle, and the use of surfactants [1,3]. Due to pre-corneal loss and these anatomical barriers, ocular absorption into the anterior chamber is typically restricted to the 3% of the instilled dose [4]. Another dynamic barrier of the cornea is the activity of transmembrane efflux pumps that restrict drug transport to the aqueous humor [5]. Conversely, a significantly larger fraction of the drug enters the systemic circulation via the conjunctival and nasal mucosae [6].

Indeed, the conjunctiva is notably more permeable than the cornea—approximately 20-fold greater for small, water-soluble molecules—owing to its leakier tight junctions and a surface area 17 times that of the cornea [6]. Specifically, the perilimbal conjunctiva provides an efficient trans-scleral route for delivering pharmacological agents to anterior segment structures, including the iris and ciliary body [6].

Delivering topical formulations to the posterior segment currently represents a major hurdle in ophthalmology. If the ocular bioavailability to the anterior segment of the eye is typically less than 5% after a single instillation of conventional eye drops, the fraction reaching the posterior segment and the retina is profoundly lower—often negligible—due to the sequential physical and anatomical and physiological barriers [7,8]. Specifically, topically administered drugs must not only overcome corneal and conjunctival clearance but also static intraocular structures, such as the lens and vitreous body, alongside restrictive biological boundaries, including the anterior blood–aqueous barrier and the posterior blood–retinal barrier (BRB) [7,8]. There are two primary penetration pathways to the posterior segment. The classical corneal route involves passage through the aqueous humor into the anterior chamber, followed by diffusion through or around the crystalline lens to reach the vitreous body and, ultimately, the retina. The primary limitation of this route is the continuous turnover of aqueous humor; consequently, the drug concentration reaching the vitreous is often negligible (approximately 1:1,000,000 of the initial dose) [9]. The conjunctival route, bypassing the anterior chamber, is considered a more promising strategy for retinal therapies. Direct diffusion occurs via the conjunctival-scleral (trans-scleral) pathway, moving through the suprachoroidal space to the retina [7]. Alternatively, the conjunctival-vascular pathway involves direct absorption into the conjunctival vasculature, which carries a higher risk of systemic distribution and associated side effects. Modern research is increasingly focused on this route to develop nanotechnologies designed to enhance molecular distribution to the posterior segment [9]. The conjunctival-vascular pathway, conversely, involves direct uptake into the conjunctival blood vessels, posing a higher risk of systemic absorption and subsequent adverse effects. Modern research is increasingly focused on this route to develop nanotechnologies designed to enhance molecular distribution to the posterior segment [9].

### 1.3. Adverse Effects and Complications

Despite its ease of administration, the topical route presents several challenges related to tolerability and side effects. Ophthalmic solutions must be formulated with a pH and tonicity closely matching those of the tear film (approximately pH 7.4) to minimize stinging, pain, and irritation, all of which significantly reduce patient compliance [1].

Furthermore, the prolonged use of eye drops—particularly those containing preservatives such as Benzalkonium Chloride (BAK)—is associated with Ocular Surface Disease (OSD) and tear film instability [1,3]. Preservatives affect ocular absorption through a short-term mechanism of permeability enhancement opening epithelial junctions, and a long-term ocular surface toxicity affecting goblet cells secretion [1]. Certain formulations with higher viscosity, such as gels or ointments, may induce ocular irritation, leading to increased lacrimation, elevated blinking frequency, and transient blurred vision [1].

### 1.4. Patient Adherence

The management of ocular diseases via topical administration is inherently linked to patient compliance, a factor that frequently represents the weakest link in the therapeutic chain. Epidemiological data highlight that adherence remains critical, with approximately 50% of patients failing to correctly adhere to medical prescriptions [10]. Forgetfulness emerges as the primary cause, further exacerbated by the burden of frequent daily instillations (4–6 times per day), a lack of perceived efficacy, and immediate post-instillation discomfort [10].

In chronic diseases such as glaucoma, suboptimal compliance is particularly evident, characterized by poor adherence, lack of persistence, and premature discontinuation of therapy [11]. Beyond forgetfulness and lack of symptomatic relief, other relevant factors include administration difficulties—often associated with physical impairments such as arthritis or hand tremors—and OSD, which is frequently induced by the eye drops themselves [12].

### 1.5. The Need for Innovative Administration Routes

In recent years, growing interest has centered on exploiting intranasal drug delivery as an alternative strategy to treat a wide spectrum of anterior and posterior segment ocular disorders, aiming to address the inherent pharmacokinetic limitations of conventional topical ophthalmic therapies [13,14,15].

Characterized by rich vascularity and an expansive absorption surface area, the nasal mucosa exhibits slower mucociliary clearance rate compared to nasolacrimal drainage. Such properties ensure an extended window for absorption, promoting efficient drug delivery to both systemic and target-specific sites [16,17].

Intranasally administered drugs bypass the corneal barrier, avoid the rapid washout of the pre-corneal tear film and eliminates the direct toxicity of preservatives on the corneal and conjunctival surfaces, enhancing long-term safety and reducing ocular irritation. Furthermore, the use of a nasal spray is a simpler and more familiar technique for patients compared to the precise coordination required for eye drop instillation [1].

Finally, the nasal route offers the potential for targeted ocular delivery through neural and vascular pathways between the nasal cavity and the orbit. By achieving therapeutic intraocular concentrations without the high risk of systemic exposure typical of oral administration, or the high invasiveness and patient discomfort associated with intravitreal injections, this route represents a promising non-invasive alternative to optimize therapeutic efficacy in chronic ocular diseases while preserving patient safety [18].

This review focuses on chronic ocular diseases rather than acute conditions. Acute pathologies, such as bacterial keratitis or acute angle-closure glaucoma, require immediate, high-concentration topical loading doses applied directly to the cornea. Pharmacokinetically, the intranasal route is unsuited for such emergency interventions due to its reliance on indirect delivery pathways. Conversely, the true value of nose-to-eye delivery lies in the long-term management of chronic conditions (e.g., glaucoma, dry eye, retinal degeneration), where the goals are sustaining steady-state tissue concentrations, maximizing patient compliance, and eliminating the cumulative toxicity of topical preservatives [1,10,11,12].

## 2. Nose-to-Eye Transport Pathways: Anatomical and Physiological Basis

While primarily known for facilitating drug transport to the Central Nervous System (CNS) through the olfactory neuroepithelium [19,20], the intranasal route is now demonstrating significant potential for ocular delivery by leveraging a complex network of physiological and anatomical interconnections.

### 2.1. The Reflex Route: The Nasolacrimal Reflex

**Figure 1:**
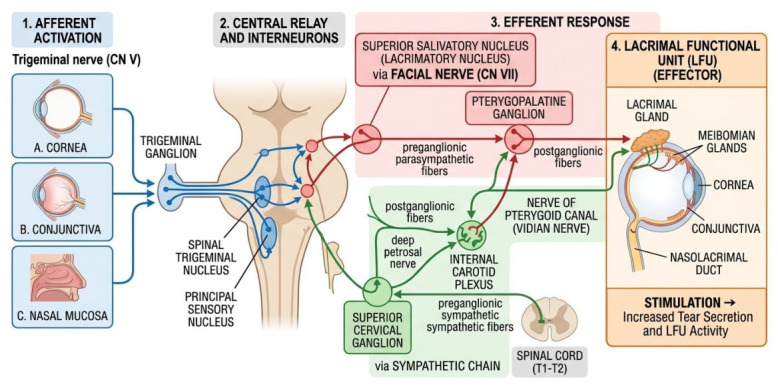
Schematic depiction of the nasolacrimal reflex.

The nasolacrimal reflex (NLR) stands out as the most extensively documented pathway and the only one to have been approved for clinical use, with varenicline nasal spray serving as the primary example of this approach [13]. Lacrimal secretion is regulated by neural reflex arcs, including those triggered by the activation of trigeminal afferent nerves in the cornea, conjunctiva, and nasal mucosa. This activation subsequently stimulates the parasympathetic and sympathetic efferent fibers of the facial nerve that innervate the Lacrimal Functional Unit (LFU) [14]. The naso-lacrimal reflex is responsible for approximately one-third of basal tear film production [14]. In this specific context, intranasally administered drugs do not physically reach the eye; instead, they activate its physiological function remotely. Recently, intranasal neurostimulation of the naso-lacrimal reflex (NLR) or the trigeminal parasympathetic pathway (TPP) has emerged as an innovative therapeutic strategy for Dry Eye Disease (DED) [13,14] (see Figure 1).

### 2.2. The Local Route: Diffusion and Perineural Transport

**Figure 2:**
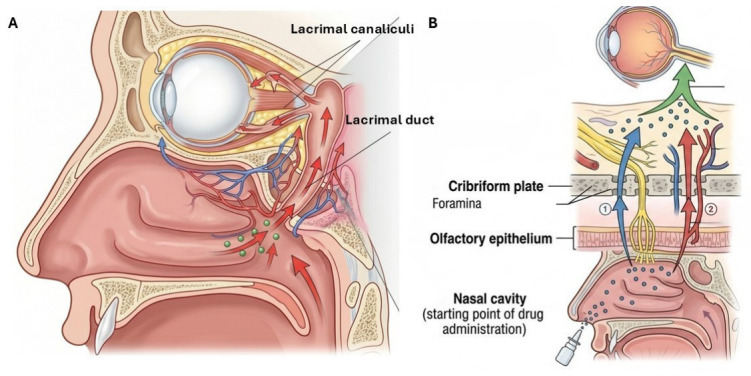
Schematic depiction of (**A**): Diffusion via the nasolacrimal duct. (**B**): Diffusion via the neural and vascular pathway; (**①**): Perineural transport, (**②**). Vascular transport.

While the bony orbit acts as a physical barrier, significant evidence suggests local communication between the nasal cavity and the eye. Pharmaceutical agents may be transported through the following pathways:

- Diffusion via the Nasolacrimal Duct (NLD): Although physiological flow is directed from the eye to the nasal cavity, it has been hypothesized that under specific formulation conditions (e.g., gels or nanoparticles), partial retrograde flow or local absorption may occur within the ductal mucosa, which communicates with the lacrimal sac [21,22] (See Figure 2A).
- Direct Neural and Vascular Pathways: Often referred to as the ‘Neural Bridge’, this route is a critical area of research for posterior segment targeting. It leverages the neural and vascular networks that traverse the cribriform plate, connecting the nasal cavity with the orbit. The olfactory epithelium is situated immediately below the cribriform plate, which separates the nasal and orbital cavities. Numerous vessels and nerves penetrate the orbit through small foramina in the cribriform plate, such as the anterior and posterior ethmoidal branches of the ophthalmic artery, which supply the olfactory epithelium [18]. Beyond this vascular link, drugs can diffuse directly along the perineural sheaths of nerves emerging from the nasal cavity. By traversing the cribriform plate, these molecules reach the orbital regions, ocular tissues, and the optic nerve, effectively bypassing the blood–retinal barrier (BRB) [23]. Evidence from radiotracer imaging in rats demonstrates that molecules traversing olfactory nerves and lymphatic channels can reach significant molecular accumulation in the optic nerve as early as 30 min post-intranasal administration [23,24]. Furthermore, the olfactory neuroepithelium is innervated by the trigeminal nerve. Its branches, specifically the ethmoidal and nasociliary nerves, provide a potential preferential and rapid transport pathway through the perineural space toward ocular structures. This is supported by evidence showing that, when administered intranasally, drug concentrations in the trigeminal and optic nerves are significantly higher than in other connected structures, such as the olfactory bulbs or the striatum. This suggests that trigeminal-innervated structures, including the eye, receive the drug directly via the nerve fibers [18] (See Figure 2B). The anatomo-functional interplay between these sensory pathways is further corroborated by clinical evidence demonstrating that glaucoma, is closely associated with measurable olfactory dysfunction [25]. This shared neurodegenerative vulnerability highlights the physiological contiguity that the nose-to-eye route exploits for drug delivery.

However, because the trigeminal nerve anatomically roots into the pons of the brainstem, this neural pathway inherently acts as a shared conduit [19,20]. Consequently, the same mechanism that facilitates localized ocular delivery simultaneously opens a direct gateway to the CNS, posing a potential risk of central translocation.

### 2.3. The Systemic Route: Indirect Uptake

**Figure 3:**
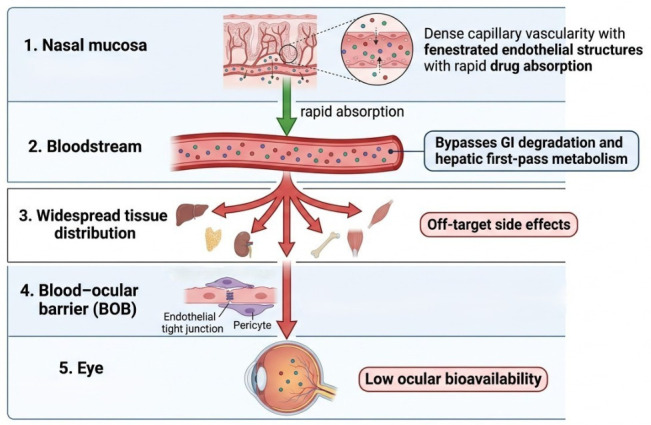
Systemic drug delivery to the eye.

Due to its dense vascularity and fenestrated endothelial structure, the nasal mucosa allows for rapid drug entry into the bloodstream [19]. This pathway offers the distinct advantage of bypassing gastrointestinal degradation and hepatic first-pass metabolism. Despite these advantages, systemic delivery to the eye is limited by the blood–ocular barrier and widespread tissue distribution through bloodstream. These factors diminish the bioavailability at the ocular site while elevating the potential for off-target side effects. Thus, the systemic route is inherently less effective than direct nasal-to-ocular transport for achieving high local drug concentrations [26] (See Figure 3).

## 3. Pre-Clinical Evidence: Animal Studies

Pre-clinical animal models have provided the first crucial evidence regarding the bioavailability and therapeutic efficacy of intranasally administered drugs targeting the ocular compartment. Research has primarily utilized murine models (mice and rats) to evaluate the intranasal delivery of various molecules—including erythropoietin (EPO), nerve growth factor (NGF), ST266, resveratrol, insulin, and epigallocatechin—typically administered as saline-based drops via micropipettes [18,23,24,27,28,29,30,31,32,33].

A significant example of the efficacy of the intranasal route is found in studies of Erythropoietin (EPO) administration in rodent models of N-methyl-N-nitrosourea-induced retinal degeneration [18]. Research demonstrates that IN application yields significantly higher retinal drug concentrations compared to intravenous (IV) or conventional topical (eye drop) administration. This approach has shown clinical efficacy in preserving photoreceptors from apoptosis [18], thereby confirming the direct transport pathway between the nasal cavity and the orbit. Furthermore, IN administration resulted in reduced erythropoietic stimulation compared to IV dosing, effectively mitigating thromboembolic side effects [18].

The efficacy of recombinant human erythropoietin (rhEPO) also validated in a rat model of chronic cerebral ischemia (CCI) induced by permanent bilateral common carotid artery occlusion [27]. This study highlights how nasal delivery allows rhEPO to bypass the blood–brain barrier (BBB), reaching the central nervous system (CNS) non-invasively while minimizing systemic exposure. Beyond cognitive improvement, the treatment produced significant benefits for visual function, as measured by flash visual evoked potentials (FVEP). These findings were supported by histological evidence confirming a reduction both in retinal thinning and in loss of retinal ganglion cells (RGCs) [27].

The IN route has been extensively explored for delivering neurotrophic agents and macromolecules that struggle to penetrate the CNS via other routes. For instance, nerve growth factor (NGF) was administered intranasally in AD11 murine models of neurodegeneration, suggesting potential neuroprotective effects [28]. Notably, a comparison between IN and topical ocular NGF delivery revealed that the former was significantly more effective in reversing disease phenotypes, whereas topical ocular administration showed limited efficacy even at high dosages [28]. This suggests that the IN route represents a superior and versatile platform for treating pathologies involving the brain–eye axis.

Furthermore, there is growing evidence of targeted intranasal applications involving neuroprotective agents specifically directed toward the posterior segment of the eye.

ST266 (an Amnion-Derived Cellular Cytokine Solution) is a biological secretome from multipotent progenitor cells that acts as a neuroprotective agent by reducing oxidative stress and mitochondrial dysfunction [23,24,29]. Studies on experimental autoimmune encephalomyelitis (EAE) murine models—serving as a proxy for multiple sclerosis-related optic neuritis—confirm that IN-delivered ST266 reaches ocular tissues in therapeutically relevant amounts. Results include preserved visual function (assessed via optokinetic response, OKR), significant reduction in RGCs degeneration, and decreased inflammation/demyelination of the optic nerve [23,24,29]. Radiolabeled molecule studies further confirm higher accumulation in the optic nerve and retina compared to systemic administration [23,24,29].

Similarly, another study compared the neuroprotective effects on RGCs of oral versus intranasal Resveratrol Nanoparticles (RNs) in EAE murine models [30]. IN treatment showed significantly higher RGCs survival rates with a much lower required concentration (up to 50% less) than oral therapy, validating the ability of the IN route to bypass the blood–brain barrier (BBB) and blood–retinal barrier (BRB) [30].

Recent research has focused on intranasal insulin as a strategy against neurodegeneration. This route allows insulin to reach the CNS and retina [31] without causing systemic hypoglycemic effects, a major challenge with systemic insulin [31,32]. While Wong et al. emphasized the “nose-to-brain” strategy for central insulin resistance in Alzheimer’s disease [31], Ong et al. extended this potential to the eye using streptozotocin-induced diabetic retinopathy rat models [32]. IN insulin helped regulate inflammatory and apoptotic pathways, preventing the thinning of the inner plexiform and ganglion cell layers while reducing pro-inflammatory cytokines and reactive gliosis [32].

An innovative frontier in retinal neuroprotection involves IN biological nanocarriers, specifically platelet-derived extracellular vesicles (PEVs), for transporting epigallocatechin gallate (EGCG). EGCG is the primary catechin found in green tea, acting as a powerful antioxidant by lowering inflammation and blocking the nuclear factor (NF)-κB signaling pathway [33]. In dexamethasone-induced glaucoma murine models, this system improved RGCs survival, preserved retinal thickness, and reduced reactive oxygen species (ROS) and pro-inflammatory cytokines, preserving visual function [33]. Fluorescent markers confirmed the preferential transport of these vesicles along the olfactory and trigeminal nerve sheaths, with rapid distribution to the optic nerve and retina [33].

## 4. Clinical Evidence: Human Applications

Translating pre-clinical findings into human validation is a critical step in defining the clinical role of intranasal route in ocular therapeutics. To date, direct clinical applications for treating ocular diseases via the nasal mucosa are still confined to advanced pre-clinical stages and ongoing clinical trials.

### 4.1. Anterior Segment and Dry Eye Disease

The evolution of research into intranasal drug delivery has led to the approval of novel therapeutic strategies that leverage neural reflexes to treat ocular surface pathologies, effectively bypassing the barriers inherent to traditional eye drops. A significant example is intranasal varenicline (OC-01) (Tyrvaya; Oyster Point Pharma, Princeton, NJ, USA), a partial nicotinic acetylcholine receptor agonist approved by the FDA in 2021 for the treatment of Dry Eye Disease (DED) [13,14].

Unlike topical therapies that primarily aim to suppress inflammation, varenicline operates by activating the trigeminal parasympathetic pathway (TPP), thereby stimulating the basal tear film production [13,14]. Its clinical efficacy in humans has been extensively documented, demonstrating a rapid, clinically significant, and safe improvement in tear production as quantified by Schirmer test scores [13,14].

### 4.2. Ocular Inflammation and Allergic Conjunctivitis

Long-standing clinical evidence suggest that corticosteroids are associated with potential ocular risks, such as glaucoma and cataract formation, particularly in oral or topical drop formulations. However, recent research has shifted the focus toward the capacity of intranasal steroids (INS) to alleviate the ocular symptoms of allergic rhinitis (AR), typically assessed through the total ocular symptom score (TOSS), which includes itching, redness and tearing [21]. Furthermore, large-scale clinical trials with modern INS (such as fluticasone furoate and mometasone furoate) have demonstrated no significant increase in the risk of glaucoma or cataracts in the general population [21].

Evidence for the ocular efficacy of INS on allergic rhinoconjunctivitis dates back to a comprehensive 1998 meta-analysis, which established their non-inferiority to oral antihistamines [34]. Consequently, INS were prioritized as a first-line treatment for AR, targeting both nasal and ocular manifestations [34]. This clinical direction has been reinforced by more recent studies confirming a significant reduction in TOSS, supporting the intranasal administration of various steroid molecules, including fluticasone propionate [35,36,37,38], fluticasone furoate [39], mometasone [40], triamcinolone acetonide [34], budesonide [41], and beclomethasone dipropionate [21,22,42].

Although the exact pathophysiological mechanisms remain to be fully elucidated, current clinical hypotheses explaining the efficacy of INS on ocular symptoms suggest a combination of the following: (1) Improved Nasolacrimal Drainage: The anti-inflammatory action of INS reduces mucosal swelling in the nasal cavity, facilitating drainage through the nasolacrimal duct, promoting tear outflow, thus reducing the residence time of allergens on the conjunctival surface. (2) Direct or Systemic Drug Transfer: Steroids may reach the eye in trace amounts either via systemic absorption or through physical retrograde flow via the nasolacrimal duct. Systemic absorption, however, appears to be the least likely contributor due to the minimal drug concentrations reaching the eye. (3) Inhibition of the Naso-Ocular Reflex: Reducing nasal inflammation modulates the trigeminal neural reflex activity triggered by allergens. (4) Systemic Immunological Effect: Following the “one airway, one disease” concept, treating the nasal mucosa may induce a systemic immune response that attenuates inflammation at distal sites, including the lungs and the eyes [21,22,40].

In conclusion, there is compelling evidence that INSs are both effective and safe for managing the nasal and ocular symptoms of allergic rhinoconjunctivitis, thereby improving patient quality of life. They maintain a favorable ocular safety profile; current literature suggests that INS used at therapeutic doses—even for several months—do not significantly increase the risk of cataract formation, IOP elevation, or glaucoma [22]. Additionally, modern compounds like fluticasone furoate and mometasone furoate exhibit negligible systemic bioavailability (<1%), further minimizing the risk of systemic adverse effects [22]. While these formulations show promising efficacy in bypassing ocular barriers, significant translational limitations and pharmacokinetic variability remain, primarily driven by inter-species anatomical differences, specific formulation properties, and patient-dependent factors. Table 1 provides a structured overview of these elements, highlighting the critical barriers that future research must overcome.

## 5. Safety: Intranasal Steroids and Intraocular Pressure

A comprehensive safety evaluation of intranasal corticosteroids (INCS) is a pivotal requirement for validating their clinical utility, particularly when positioning this route as a viable alternative to traditional topical ophthalmic administration. Specifically, corticosteroid-induced elevation of intraocular pressure (IOP) represents one of the most complex clinical challenges in managing chronic ocular diseases. However, IN administration introduces a distinct pharmacokinetic variable: the ability to deliver therapeutic effects while potentially reducing the direct exposure of anterior segment tissues to the high concentrations typical of eye drops. Consolidated evidence from two major global meta-analyses (Valenzuela et al., 2019 [43]; Donaldson et al., 2020 [44]) provides a reassuring outlook regarding the long-term tolerability and safety of INS in adults. No significant ocular changes are associated with the use of INS—whether administered as sprays, drops, aerosols, or irrigations—specifically concerning IOP [43,44], glaucoma development [43], and cataract formation [43]. Unlike oral or topical ophthalmic corticosteroids, which carry a well-documented risk of ocular hypertension, intranasal corticosteroids act locally with minimal systemic absorption. While the ocular safety profile is excellent, clinical surveillance should instead focus on local tolerability. Epistaxis emerges as the most common adverse event, with a significantly higher risk compared to placebo, particularly when the drug is administered as drops rather than sprays [44]. Conversely, non-specific symptoms such as headaches have shown no direct correlation with steroid treatment. On a systemic level, the function of the hypothalamic–pituitary–adrenal (HPA) axis remains preserved in the vast majority of patients (approximately 78% of studied cases) [44].

A major literature review acknowledges that prolonged use of inhaled or intranasal glucocorticoids for respiratory disorders may cause ocular hypertension (OHT) in a small subset of susceptible patients [45]. The hypothesized causes include systemic drug absorption or improper administration techniques leading to direct ocular contact [45]. Some studies report OHT or steroid-induced glaucoma (SIG) in patients using high doses of fluticasone for extended periods, but almost exclusively in those with a first-degree relative affected by glaucoma [45]. In contrast, studies on populations with chronic obstructive pulmonary diseases have found no association between INS and glaucoma, either in healthy subjects or in patients with pre-existing ocular hypertension. Research focusing on intranasal steroids for rhinitis and upper airway treatment yields highly reassuring results, showing no increase in IOP even in patients with controlled primary open-angle glaucoma (POAG) [45].

In conclusion, while the overall risk remains low, clinicians are advised to monitor patients on long-term therapy, especially those with a family history of glaucoma or those identified as “steroid responders” [45].

## 6. Advantages and Limitations of the Intranasal Route

Intranasal drug delivery represents a developing therapeutic frontier in ophthalmology, offering a strategic shift from conventional methods. It effectively addresses the low bioavailability inherent to anterior segment topicals, while providing a non-invasive alternative to posterior segment procedures, such as intravitreal injections, thereby circumventing their associated clinical risks.

### 6.1. Advantages

The primary strength of this route lies in its ability to bypass the limitations of low pre-corneal bioavailability and corneal tissue barrier. Instead, it leverages the highly vascularized nasal mucosa, which offers a large absorption surface area and a mucociliary clearance rate that is slower than lacrimal drainage [16,17,19].

A key value proposition of the intranasal route is its unique transport mechanism, capable of circumventing the blood–brain and blood–retinal barriers. On one hand, the rapid systemic absorption through respiratory mucosa avoids hepatic first-pass metabolism, ensuring plasma concentrations sufficient to reach the posterior segment [19]. On the other hand, a direct connection is established through perineural transport via the olfactory and trigeminal nerves [13,14,18,23].

From a clinical practice perspective, this route ensures high patient compliance by addressing two major issues. It eliminates the mechanical and chemical stress on the ocular surface often caused by chronic topical therapies and exposure to cytotoxic preservatives. It offers a painless, risk-free alternative to invasive intraocular procedures [1,18].

### 6.2. Limitations

Despite its potential, the efficacy of the nasal route is strictly conditioned by intrinsic physiological limits. Mucociliary clearance remains the primary obstacle, drastically reducing the drug’s residence time on the mucosa and limiting absorption. Nasal defense mechanisms typically move non-adherent formulations toward the nasopharynx within approximately 15–20 min [46].

Furthermore, the nasal cavity can only accommodate extremely small volumes of formulation, necessitating high-potency molecules or highly concentrated delivery systems. Other critical factors include the presence of local proteolytic enzymes, which may degrade protein-based drugs; the poor permeability for large or highly polar molecules [46]; the individual conditions such as rhinitis, nasal polyposis, congestion, or septal deviations, that can lead to an unpredictable pharmacokinetic profile [47]. Lastly, chronic use requires careful monitoring of local tolerability to prevent inflammation or anosmia. Data from adverse event reporting systems suggest that topical administration of intranasal drugs, e.g., beta blockers, can lead to numerous symptoms, ranging from localized irritation and epistaxis to more complex sensory alterations like anosmia or systemic reactions like dyspnea and dizziness [48].

In summary, while the intranasal route is a validated pathway for ocular drug delivery and offers an excellent safety profile, its therapeutic success depends heavily on the development of advanced delivery systems (e.g., nanoparticles or in situ gels). These technologies are essential to counteract mucociliary clearance and extend the nasal residence time, thereby maximizing the window for drug absorption [3,19,26].

## 7. The Role of Nanotechnology

The clinical translation of both topical ocular and intranasal drug delivery is constrained by anatomical and physiological clearing mechanisms. In this landscape, nanotechnology represents a paradigm shift, overcoming the limitations of both routes.

### 7.1. Overcoming Topical Ocular Limitations

Conventional topical eye drops fail primarily due to rapid nasolacrimal drainage, blinking, and the highly selective corneal epithelial barrier, resulting in low bioavailability [2,8,49]. In chronic ocular diseases such as glaucoma, this low bioavailability, combined with the need for frequent daily dosing, significantly reduces patient compliance and compromises therapeutic outcomes [49]. Nanocarriers address these hurdles through specific physicochemical mechanisms. Nano-sized delivery systems (such as liposomes, polymeric nanoparticles, and solid lipid nanoparticles) can fluidize or modulate the tight junctions of the corneal epithelium, allowing enhanced paracellular or transcellular transport of poorly permeable drugs [9,49]. By functionalizing nanocarriers with mucoadhesive polymers (e.g., chitosan, hyaluronic acid), the particles form hydrogen bonds with the ocular mucin layer, counteracting mechanical wash-out and extending the window for trans-scleral or corneal absorption [8,15].

### 7.2. Mitigating Nasal and Nose-to-Brain Disadvantages

While the intranasal route circumvents the corneal barrier and avoids hepatic first-pass metabolism, it faces limitations: rapid mucociliary clearance, a tiny administration volume capacity [46], and the risk of unintended, off-target CNS translocation via shared neural pathways [20,50]. Nanotechnology resolves these issues through engineered smart-delivery properties. Specifically, loading ophthalmic drugs into mucoadhesive nanocarriers or smart in situ nano-gels (which undergo a phase transition from liquid to gel upon contact with the nasal temperature or ions) significantly enhances nasal residence time [51,52], structuring a continuous drug reservoir that prevents rapid clearance into the nasopharynx [30]. Furthermore, encapsulation within these nanocarriers provides a protective shield against the proteolytic enzymes rich in the nasal mucosa, preventing the premature degradation of biopharmaceuticals, such as proteins or neurotrophic factors, and ensuring that intact macromolecules reach the target site [33,50]. Finally, to control ocular targeting versus central translocation and minimize the risk of unintended nose-to-brain delivery, nanocarriers can be engineered for controlled, localized release; by tailoring their surface charge, lipid composition, or PEGylation, researchers can favor vascular absorption through the fenestrated respiratory epithelium toward the orbit, while simultaneously limiting direct axonal internalization by the olfactory or trigeminal neurons, thereby reducing off-target brain exposure [20,51].

## 8. Conclusions and Future Perspectives

The intranasal route of administration represents a highly compelling investigational avenue within the modern ophthalmic landscape. Although numerous pre-clinical studies have successfully demonstrated the efficacy of various molecules in reaching ocular tissues and producing therapeutic effects in animal models [18,23,24,27,28,29,30,31,32,33], a substantial translational gap remains. At present, the FDA approval of varenicline nasal spray for dry eye disease stands as the only successful clinical translation in humans [13,14], whereas drug delivery to the posterior segment and the retina remains experimental.

Moving forward, it is essential to prioritize rigorous, large-scale phase 2 and 3 clinical trials to determine whether the promising results seen in rodents can be replicated in humans. These studies are essential to validate efficacy and safety profiles, confirm the ability of novel formulations to overcome human physiological barriers, and establish standardized dosing protocols. In addition, future trials must monitor for potential central side effects arising from unintended nose-to-brain translocation via shared neural pathways, as well as systemic adverse effects triggered by non-productive drug absorption through the highly vascularized nasal respiratory mucosa.

Future perspectives for intranasal delivery in ophthalmology are intrinsically linked to advancements in materials science and engineering, particularly nanotechnology. The encapsulation of ophthalmic drugs into surface-engineered nanocarriers and stimuli-responsive in situ nano-gels is essential to overcome rapid mucociliary clearance and enzymatic degradation. Furthermore, precisely tailoring the physicochemical properties of these nanoparticles (such as surface charge and PEGylation) will be crucial to maximize direct microvascular transport toward the orbital tissues while minimizing off-target systemic wash-out and direct axonal internalization by olfactory and trigeminal neurons. Alongside these technological shifts, refining delivery devices is necessary to ensure consistent dosing, independent of the patient’s inhalation technique or anatomical variability.

In summary, the topical ocular route will undoubtedly remain the preferred and standard approach for treating diseases of the anterior segment, owing to its localized effect, immediacy, and ease of administration. The intranasal route should not be viewed as a total replacement for conventional eye drops, but rather as a specialized, complementary strategy. Ultimately, establishing this route as a viable option for targeting chronic ocular pathologies requires a thorough, evidence-based validation of both its localized pharmacokinetic efficiency and its systemic safety profile.

## Figures and Tables

**Table 1:** Comparative analysis of intranasal drug delivery strategies for ocular targeting: architectural features, pharmacokinetic outcomes, and translational bottlenecks.

Drug/Molecule	Study Type	Target Ocular Segment	Formulation/Vehicle	Key Pharmacokinetic Outcome/Efficacy	Identified Translational Limitations and Variability
EPO/rhEPO	Preclinical (Rodents–Mice and Rats)	Posterior (Retina)	Intranasal aqueous solution (PBS)	High retinal concentrations vs. IV route; reduced photoreceptor apoptosis and RGC loss; recovery of visual signal (ERG and FVEP).	Anatomical differences (nasal cavity human vs. rodent). Disease kinetic mismatch in animal models. Need for precise dose translation to avoid systemic erythropoiesis/hematocrit increase.
NGF	Preclinical (AD11 Mice)	Posterior (Brain–Eye Axis)	Intranasal aqueous solution	Reversal of neurodegenerative phenotypes; significantly higher efficacy compared to topical ocular route, which showed limited efficacy even at high dosages.	Use of specific transgenic AD11 mouse models limits human translatability. High vulnerability of large proteins to nasal enzymatic degradation and mucociliary clearance. Absence of head-to-head studies using protective nanotechnological platforms.
ST266 (Amnion-derived Biological Secretome)	Preclinical (EAE Mice–MS/Optic Neuritis model)	Posterior (Retina/Optic Nerve)	Biological solution (Secretome)/Intranasal aqueous solution	Accumulates rapidly in target tissues (1.1% in optic nerve, 0.9% in vitreous). Preservation of visual function (OKR); reduced RGC loss, optic nerve inflammation, and demyelination.	High molecular complexity makes elucidating specific mechanisms of action difficult. Neuroprotection requires the full complement of proteins (>50 kDa cannot be excluded). Need for continuous daily administration to sustain long-term benefits. Unclear if drug distribution kinetics are identical in humans.
Resveratrol (Nanoparticles–TPGS/Solutol)	Preclinical (EAE Mice–MS/Optic Neuritis model)	Posterior (Retina/RGCs)	Freeze-dried polymeric nanoparticles in aqueous solution	Significant RGC survival at halved dosages (8.44 mg/kg IN vs. 16.9 mg/kg Oral). Neuroprotection occurs independently of anti-inflammatory or anti-demyelinating effects.	Fails to significantly prevent visual function decline (OKR) despite RGC survival. Human olfactory region is proportionally much smaller than in rodents, limiting CNS/retinal penetration. Nasal mucosa biochemical barriers (p-glycoprotein, MRP1, enzymes). Max dosage is strictly limited by solubility within the small maximal nasal volume capacity.
Insulin (e.g., Humulin R)	Preclinical (Transgenic db/db Mice–T2DM/DR model)	Posterior (Retina)	Intranasal aqueous solution	Reaches retina without systemic hypoglycemia. Prevents functional decline (ERG b-waves and oscillatory potentials). Reduces outer retinal thinning, reactive gliosis (GFAP), apoptosis (Caspase-3), and pro-inflammatory gene expression.	Fails to prevent inner retinal thinning (the earliest neurodegenerative event in DR). High vulnerability of naked insulin to nasal peptidases and mucociliary clearance. Primary nose-to-brain translocation requires strict monitoring of CNS side effects. Needs advanced delivery devices/nanocarriers for human translation.
EGCG	Preclinical (Dexamethasone-induced Glaucoma Mice)	Posterior (Retina/Optic Nerve)	Platelet-derived extracellular vesicles (PEVs)	Preferential transport along olfactory and trigeminal nerve sheaths with rapid distribution to optic nerve and retina. Enhanced RGC survival, preserved retinal thickness, reduction in ROS and pro-inflammatory cytokines (NF-κB pathway blockade), and preservation of visual function.	Complexity of scaling up biological nanocarriers for clinical use. Risk of unintended “nose-to-brain” translocation (off-target CNS delivery) due to shared olfactory/trigeminal neural pathways.
Varenicline (OC-01/Tyrvaya)	Clinical Human (FDA Approved–Phase II/III Trials: ONSET-1/2, MYSTIC)	Anterior (Ocular Surface/Lacrimal Functional Unit)	Preservative-free aqueous nasal spray (low-volume 0.05 mL)	Rapid (5 min) and sustained (12 weeks) increase in endogenous basal tear production (Schirmer test). Activation of the NLR/trigeminal parasympathetic pathway.	Extremely high incidence of non-ocular reflex adverse events (transient sneezing in >82% of patients, cough, throat irritation). Need for long-term real-world effectiveness data in diverse populations.
Intranasal Corticosteroids (e.g., Fluticasone, Mometasone)	Clinical Human (Systematic Reviews and Meta-analyses/Large-scale RCTs)	Anterior (Ocular Symptoms of Allergic Rhinoconjunctivitis)	Aqueous nasal spray/Aerosol/Drops	Significant reduction in TOSS equivalent to oral antihistamines. Negligible systemic bioavailability (<1% for newer INS).	Significantly increased risk of epistaxis vs. placebo (especially with drops). Although large meta-analyses show no significant IOP elevation or cataracts, monitoring is still advised for “steroid responders” or patients with a family history of glaucoma. Risk of HPA axis suppression if co-administered with inhaled steroids.

EPO/rhEPO: Erythropoietin; PBS: Phosphate-Buffered Saline; RGC: retinal ganglion cells; ERG: electroretinogram; FVEP: flash visual evoked potentials; NGF: Nerve Growth Factor; EAE: Experimental autoimmune encephalomyelitis; MS: multiple sclerosis; OKR: Optokinetic response; TPGS: D-α-tocopherol polyethylene glycol 1000 succinate; CNS: central nervous system; MRP1: multidrug resistance-associated protein; T2DM: type 2 diabetes mellitus; DR: diabetic retinopathy; GFAP: Glial fibrillary acidic protein; EGCG: Epigallocatechin Gallate; ROS: reactive oxygen species; PEVs: platelet-derived extracellular vesicles; NLR: naso lacrimal reflex; RCT: randomized controlled trial; TOSS: Total Ocular Symptom Score; INS: intranasal steroids; IOP: intraocular pressure; HPA: hypothalamic–pituitary–adrenal axis.

## Data Availability

No further data is available.

## Abbreviations

The following abbreviations are used in this manuscript:

IN	Intranasal
μL	Microliters
BRB	Blood–retinal barrier
BAK	Benzalkonium Chloride
OSD	Ocular Surface Disease
CNS	Central Nervous System
NLR	Nasolacrimal Reflex
LFU	Lacrimal Function Unit
TPP	Trigeminal parasympathetic pathway
DED	Dry Eye Disease
NLD	Nasolacrimal Duct
EPO	Erythropoietin
NGF	Nerve Growth Factor
IV	Intravenous
rhEPO	Recombinant human erythropoietin
CCI	Chronic Cerebral Ischemia
BBB	Blood–Brain Barrier
FVEP	Flash visual evoked potentials
RGCs	Retinal Ganglion Cells
EAE	Experimental autoimmune encephalomyelitis
OKR	Optokinetic response
RNs	Resveratrol Nanoparticles
EGCG	Epigallocatechin gallate
PEVs	Platelet-derived extracellular vesicles
NF-kB	Nuclear factor-kappa B
ROS	Reactive oxygen species
OC-01	Investigational code for varenicline nasal spray.
FDA	Food and Drug Administration
INS	Intranasal Steroids
AR	Allergic rhinitis
TOSS	Total ocular symptom score
INCS	Intranasal Corticosteroids
IOP	Intraocular Pressure
HPA	Hypothalamic–pituitary–adrenal
OHT	Ocular hypertension
SIG	Steroid induced glaucoma
POAG	Primary open-angle glaucoma
